# Evidence for metal inhibition of tumour membrane-bound neutral protease and the control of tumour-induced target cell cytolysis.

**DOI:** 10.1038/bjc.1982.304

**Published:** 1982-12

**Authors:** F. S. Steven, T. P. Hulley, M. M. Griffin, S. Itzhaki

## Abstract

Previous studies have characterized the enzymatic properties and inhibition of a trypsin-like neutral protease on the surface of Ehrlich ascites cells by means of kinetic analysis. The present study links these kinetic studies with the recently reported role of a tumour-cell membrane-bound serine protease in tumour-induced target cell lysis. Low-mol.-wt inhibitors of this cell-surface trypsin-like neutral protease exhibited a corresponding ability to prevent tumour-induced haemolysis. High-mol.-wt inhibitors of trypsin in free solution had no inhibitory action either on the tumour-bound enzyme or on the ability of tumour cells to lyse erythrocytes. Fragments of tumour-cell membrane retain both the trypsin-like neutral protease activity and the ability for haemolysis. This study represents a correlation between an easily assayed membrane-bound enzyme on tumour cells and a function of possible biological relevance.


*Br. J. Cancer* (1982) **46**, 934

### EVIDENCE FOR METAL INHIBITION OF TUMOUR MEMBRANE-BOUND NEUTRAL PROTEASE AND THE CONTROL OF TUMOUR-INDUCED TARGET CELL CYTOLYSIS

F. S. STEVEN, T. P. HULLEY, M. M. GRIFFIN AND S. ITZHAKI

*From the Department of Biochemistry, Stopford Building, University of Manchester, Manchester M13 9PT*

Received 7 June 1982 Accepted 24 August 1982

**Summary.—Previous studies have characterized the enzymatic properties and inhibition of a trypsin-like neutral protease on the surface of Ehrlich ascites cells by means of kinetic analysis. The present study links these kinetic studies with the recently reported role of a tumour-cell membrane-bound serine protease in tumour-induced target cell lysis. Low-mol.-wt inhibitors of this cell-surface trypsin-like neutral protease exhibited a corresponding ability to prevent tumour-induced haemolysis. High-mol.-wt inhibitors of trypsin in free solution had no inhibitory action either on the tumour-bound enzyme or on the ability of tumour cells to lyse erythrocytes. Fragments of tumour-cell membrane retain both the trypsin-like neutral protease activity and the ability for haemolysis. This study represents a correlation between an easily assayed membrane-bound enzyme on tumour cells and a function of possible biological relevance.**

EARLIER STUDIES with Ehrlich ascites tumour cells grown in mice (Steven *et al.*, 1980; Short *et al.*, 1981) demonstrated that these cells possessed a trypsin-like neutral protease located on the cell surface. This neutral protease was further demonstrated to be protected from the approach of high-mol.-wt inhibitors of trypsin but was inhibited by peptides derived by pronase digestion of these high-mol.-wt inhibitors (Steven & Griffin, 1982). It was concluded that low-mol.-wt reagents which specifically inhibited this neutral protease on the surface of tumour cells might be capable of inhibiting the tumourigenic activity of such cells (Steven & Griffin, 1982). It has recently become possible to test such a theory, employing the tumour-induced erythrocyte-cytolysis assay introduced by DiStefano *et al.* (1982). These authors reported a membrane-bound trypsin-like enzyme on the surface of Walker 256 tumour cells which caused haemolysis of erythrocytes at 37°C after a lag period of 6 h in which no lysis was observed. The activity of this protease was much higher in a preparation of tumour-cell membranes than in preparation of intact tumour cells. This observation was in contrast to an earlier report by Zucker & Lysik (1977) in which it was claimed that tumour-mediated cytotoxicity of erythroblasts required intact tumour cells. Our results clearly show that the presence of intact viable tumour cells is not a necessary requirement for target-cell lysis. Washed fragments of tumour-cell membrane are sufficient *perse* to promote target-cell lysis. All that the intact tumour cell provides is a suitably located neutral protease on its plasma membrane. Questions relating to the viability of the tumour cell and the possible effect of inhibitors used in these studies on the tumour-cell DNA can be disregarded as irrelevant to a discussion of control of tumour-cell-induced lysis of target cells, since fragments of tumour-cell membranes can replace intact viable cells in this assay.

We employed a similar haemolytic assay system, replacing the Walker 256 cells with mouse Ehrlich ascites tumour cells in the presence of incremental additions of reagents which had previously been shown to inhibit the cell-surface neutral protease (Short *et al.*, 1981; Steven *et al.*, 1982). Our results indicate that metal-ion inhibition of the neutral protease on the tumour cells corresponds to a similar inhibition of haemolysis by tumour cells. Our evidence also indicates that the cell-surface neutral protease is directly responsible for the observed haemolysis and that there is no requirement for the release of soluble proteases from the tumour cell to promote lysis.

### MATERIALS AND METHODS

Ehrlich ascites tumour cells were grown in mice (Itzhaki, 1972) and harvested after 8 days. The ascitic plasma was removed by centrifugation and the cells ($\geqslant 95\%$ of the cells being tumour cells) washed 3 × in a large excess of physiological saline and centrifuged to give a pellet of washed cells. The washed tumour cells were resuspended in 0·9% NaCl and a cell count performed. The volume of fluid containing tumour cells was then adjusted to provide a suspension of cells having $\sim 5 \times 10^8$ cells/ml. A preparation of cell-membrane fragments was made by freezing washed tumour cells followed by thawing and sonicating in 0·9% NaCl. The sonicated cells membranes were then centrifuged on a bench centrifuge, 100 *g*, for 5 min to remove intact cells; the supernatant fraction containing the disrupted cell membranes was then centrifuged at 120,000 *g* for 1 h to collect a pellet of cell-membrane fragments. The pellet was washed by centrifugation in 0·9% NaCl 2 × and finally a suspension of membrane fragments in NaCl containing 30 mg protein/ ml was prepared.

Fresh normal human blood was suspended without anticoagulant in a large volume of 0·9% NaCl and rapidly centrifuged to remove plasma constituents. The erythrocytes were carefully washed 3 × in 0·9% NaCl and finally suspended in a medium containing 2mM glucose dissolved in 0·9% NaCl. The erythrocyte count of this suspension was adjusted to provide $\sim 10^9$ cells/ml. Fresh human serum was also obtained from these donors.

Aprotinin supplied as the drug trasylol (10,000 k.i.u./ml) was kindly provided by Bayer. The gold-containing anti-rheumatic drug Auranofin used in this study was donated by Smith, Kline and French Ltd; this was dissolved in water to give a stock solution containing 10 mg Auranofin/ml. The chemical structure of Auranofin is S-(triethylphosphoranediylaurio)- 1 -thio - β - D - glucopyranose 2,3,4,6-tetra-acetate.

All reactions were carried out in screw-capped plastic bijou tubes. In each tube was placed 0·5 ml tumour-cell suspension or cell-membrane fragments, 2 ml of 2mM glucose–NaCl, plus 0·5 ml erythrocyte suspension. The incremental addition of potential inhibitors was made with a 0–100μl microsyringe. The contents of the tubes were mixed and allowed to stand without shaking at 37°C for 18 h.

The contents of the tubes were then stirred and centrifuged for 3 min at 200 *g*. The release of haemoglobin from the erythrocytes was measured by withdrawing 100μl aliquots from the clear supernatant fractions of each tube and placing this sample in 3 ml 0·9% NaCl before spectrometric analysis at 530 nm, the isosbestic point of haemoglobin and methaemoglobin (Joiner & Lauf, 1978). The total haemoglobin content of the erythrocytes was determined on control tubes to which 100 μg of saponin had been added. We decided to arrange conditions so that the added tumour cells caused between 30 and 40% total haemolysis. The inhibition of tumour-cell-induced haemolysis is presented as a percentage of the effective haemolysis by tumour cells in the absence of potential inhibitors. For example, if the tumour cells caused 38% haemolysis, then we would give this control the value of 100 on the vertical scale of Figs 1–3, whilst the horizontal axis defines the final concentration of potential inhibitor in the test system, having a total volume of 3 ml. In order to demonstrate that the intact living tumour cells were not a requirement for target-cell cytolysis, we employed a similar incremental addition of cell-membrane fragments to erythrocytes in 3 ml 0·9% NaCl containing 2mM glucose and incubated this mixture for 18 h at 37°C. Haemolysis was measured as described above, and in this case the degree of haemolysis (Fig. 4) is presented as a percentage of the total haemolysis obtained by adding 100 μg of saponin to the control tubes in which no tumour-cell fragments were included.

The viability of the tumour cells in 0·9% NaCl containing 2mM glucose and incubated for 2 h at 37°C in the presence of potential inhibitors of target cell cytolysis was determined by the exclusion of trypan blue.

### RESULTS

The results presented in Figs 1–3 were taken in each case from a single experimental run. The data were obtained from 24 individual tubes which constitute an experimental run and contained (i) control tubes with no tumour cells, (ii) control tubes with tumour cells plus erythrocytes, (iii) control tubes with potential inhibitor and erythrocytes, and (iv) tubes with tumour cells plus incremental additions of potential inhibitor plus erythrocytes. For each potential inhibitor (*e.g.* Auranofin, etc.), 4 separate experimental runs were performed with fresh tumour cells and erythrocytes. The data obtained from each of these was plotted as an inhibition graph, only 1 of which is presented in Figs 1–3, as being representative of the 4. The 4 graphs closely approximated (within ±5%) to that presented in each figure.

The viability of control tumour cells after 2h incubation was of the same order as the viability of tumour cells in the presence of potential inhibitors as shown in the Table.

Fresh human serum (up to 200 μl/tube or 66 μl/ml) and aprotinin (up to 200 μl/tube, or 2000 k.i.u./tube) had no inhibitory action on the haemolytic activity of Ehrlich ascites tumour cells (Fig. 1, dotted line). Low-mol.-wt inhibitors of the tumour-cell-surface neutral protease, *e.g.* Auranofin and zinc ions, exhibited a marked inhibitory action on tumour-induced haemolysis of erythrocytes (Figs 1 & 2). In these experiments the pH remained virtually constant throughout (pH 6·3) and only 1–2% of the erythrocytes lysed in the absence of tumour cells. When free trypsin (20 μg/tube) was added to the controls containing no tumour cells, no haemolysis was demonstrated.

When cell-membrane fragments were employed in place of intact viable tumour cells, target-cell lysis could readily be demonstrated (Figs 3 & 4). Lysis induced by tumour-cell-membrane fragments was also inhibited by Auranofin and $ZnSO_4$; the data for $ZnSO_4$ are presented in Fig. 3. In order to demonstrate that lysis was independent of the presence of viable tumour cells, we employed incremental

TABLE—*Viability of tumour cells after 2 h at 37°C assayed by trypan-blue uptake*

| Test systems | Inhibitor | % Viability* |
|---|---|---|
| Control tumour cells | — | 55 |
| Tumour cells plus | 50 μM Auranofin | 55 |
| Tumour cells plus | 100 μM Auronofin | 55 |
| Tumour cells plus | 150 μM Auronofin | 55 |
| Tumour cells plus | 0·5 mM $ZnSO_4$ | 67 |
| Tumour cells plus | 1·0 mM $ZnSO_4$ | 54 |
| Tumour cells plus | 0·5 mM $ZnSO_4$ | 56 |
| Tumour cells plus | 3·0 mM $ZnSO_4$ | 53 |
| Tumour cells plus | 3·5 mM $ZnSO_4$ | 59 |

* Expressed as % of total cells counted.

FIG. 1.—*Haemolysis by tumour cells in the presence of serum, aprotinin and Auranofin.* Nonspecific inhibitors of proteases such as human serum and aprotinin has no effect on the rupture of erythrocytes by tumour cells (dotted line). Auranofin, capable of inhibiting tumour-cell-surface neutral protease, was an effective inhibitor of erythrocyte lysis by added tumour cells (curve).

FIG. 2.—*Haemolysis by tumour cells in the presence of zinc ions.* Zinc ions which inhibit the tumour-cell surface neutral protease also inhibit erythrocyte lysis by added tumour cells.

FIG. 3.—*Haemolysis by tumour-cell-membrane fragments in the presence of zinc ions.* Zinc ions which inhibit the tumour-cell-membrane-bound neutral protease also inhibit the lysis of erythrocytes by added tumour-cell-membrane fragments (15 mg/tube).

additions of tumour-cell-membrane fragments to a fixed number of erythrocytes and showed a direct relationship between the degree of haemolysis and the quantity of washed membrane protein added to the system (Fig. 4).

### DISCUSSION

Although we report the viability of tumour cells under our experimental conditions, the data in Figs. 3 & 4 make the discussion of cell viability irrelevant. The results clearly indicate that high-mol.-wt inhibitors of proteolytic enzymes, *viz.* serum proteins, rich in $\alpha_2$-macroglobulin and 6 other trypsin-like enzyme inhibitors (Heimburger, 1975) failed to inhibit tumour-induced haemolysis as would be predicted from our earlier studies. Similarly the reagent aprotinin (mol. wt ~12,000; Rifkin & Crowe, 1977), with a wide specificity for proteolytic enzymes, failed to inhibit tumour-cell-induced haemolysis. Neither serum nor aprotinin is an effective inhibitor of the cell-surface trypsin-like enzyme of Ehrlich ascites tumour cells (Steven *et al.*, 1980; Steven & Griffin, 1982). Free trypsin was unable to cause haemolysis, which may also indicate that enzymes in free solution are not responsible for the observed haemolysis.

Metal ions, which have been shown to be good inhibitors of cell-bound neutral proteases on tumour and sperm cells (Short *et al.*, 1981; Steven *et al.*, 1982) were good inhibitors of tumour-induced haemolysis (Figs 1–3). It should be pointed out that direct comparisons cannot be made between the molarity of metal ions required to cause inhibition of tumour-cell-surface $\beta$-naphthylamidase activity (Short *et al.*, 1981) and inhibition of tumour-mediated haemolysis, since the erythrocytes will also bind metal ions and alter the effective ionic concentration in the test system. It is clear, however, that good

FIG. 4.—*Haemolysis by incremental additions of tumour-cell-membrane fragments.* The data show that the haemolytic neutral protease is located on the tumour-cell membrane and that intact viable tumour cells are not necessary for erythrocyte lysis.

inhibitors of the cell-surface β-naphthylamidase activity were also good inhibitors of tumour-mediated haemolysis.

The data obtained with tumour-cell-membrane fragments (Figs 3 & 4) confirm the claim by DiStefano *et al.* (1982) that the effective proteolytic enzyme for target-cell lysis is located on the tumour-cell membrane. It is not necessary to employ intact tumour cells to observe target-cell cytolysis. It can be concluded that since cytolysis induced by tumour-cell-membrane fragments (Fig. 3) can be inhibited by $Zn^{++}$, etc., these inhibitors control this enzyme directly, rather than by influencing the tumour-induced cytolysis through modification of tumour DNA.

$Zn^{++}$ is almost equally as effective in inhibiting haemolysis by the membrane fragments (Fig. 3) and by intact tumour cells (Fig. 2).

We believe the results presented above strengthen the claim made by DiStefano *et al.* (1982) that tumour-induced target-cell lysis is mediated by a membrane-bound protease which is similar to trypsin and which may be assayed by the fluorescent β-naphthylamidase assay (Short *et al.*, 1981). This study therefore links the previously reported kinetic studies (Steven *et al.*, 1980; Short *et al.*, 1981) of the tumour-cell-surface neutral protease and the study of its inhibition, with one of the observable biological properties of tumour cells, namely target cytolysis (DiStefano *et al.*, 1982). We believe further studies of the selective inhibition of this important cell-surface neutral protease could lead to further developments in the control of the biological properties of tumour cells, for example cell lysis and the generation of proteolytic enzymes of crucial importance in the pathophysiology of cancer invasion.

We wish to thank Dr William Ofosu-Appiah for his skilful and painless removal of our blood samples on almost every day of the last 4 months. Without this supply of basic raw materials our study could not have been attempted.

### REFERENCES

DISTEFANO, F. J., BECK, G., LANE, B. & ZUCKER, S. (1982) Role of tumour cell membrane-bound serine proteases in tumour-induced target cell cytolysis *Cancer Res.*, **42**, 207.

HEIMBURGER, N. (1975) Proteinase inhibitors of human plasma, their properties and control functions. In *Proteases and Biological Control* (Ed. Rich *et al.*) USA Cold Spring Harbor Lab. p. 367.

ITZHAKI, S. (1972) Regulation for RNA synthesis in Ehrlich ascites tumour cells by glucose metabolism' *Life Sci.*, **11** (Part II), 649.

JOINER, C. H. & LAUF, P. K. (1978) The correlation between ouabain binding and potassium pump inhibition in human and sheep erythrocytes. *J. Physiol.*, **283**, 155.

RIFKIN, D. B. & CROWE, R. M. (1977) Isolation of a protease inhibitor from tissues resistant to tumour invasion. *Hoppe-Seyler's Z. Physiol. Chem.*, **358**, 1525.

SHORT, A. K., STEVEN, F. S., GRIFFIN, M. M. & ITZHAKI, S. (1981) β-naphthylamidase activity of the cell surface of Ehrlich ascites cells, reversible control of enzyme activity by metal ions and thiols. *Br. J. Cancer.*, **44**, 709.

STEVEN, F. S. & GRIFFIN, M. M. (1982) Inhibition of free and bound trypsin-like enzymes. *Eur. J. Biochem.*, **126**, 311.

STEVEN, F. S., GRIFFIN, M. M. & CHANTLER, E. N. (1982) Inhibition of human and bovine sperm acrosin by divalent metal ions. Possible role of zinc as a regulator of acrosin activity. *Int. J. Androl.*, **5**, 401.

STEVEN, F. S., GRIFFIN, M. M., ITZHAKI, S. & AL-HABIB, A. (1980) A trypsin-like neutral protease on Ehrlich ascites cell surfaces, its role in the activation of tumour-cell zymogen of collagenase. *Br. J. Cancer*, **42**, 712.

ZUCKER, S. & LYSIK, R. (1977) Cancer induced cytolysis of normal bone marrow cells. *Nature*, **265**, 736.


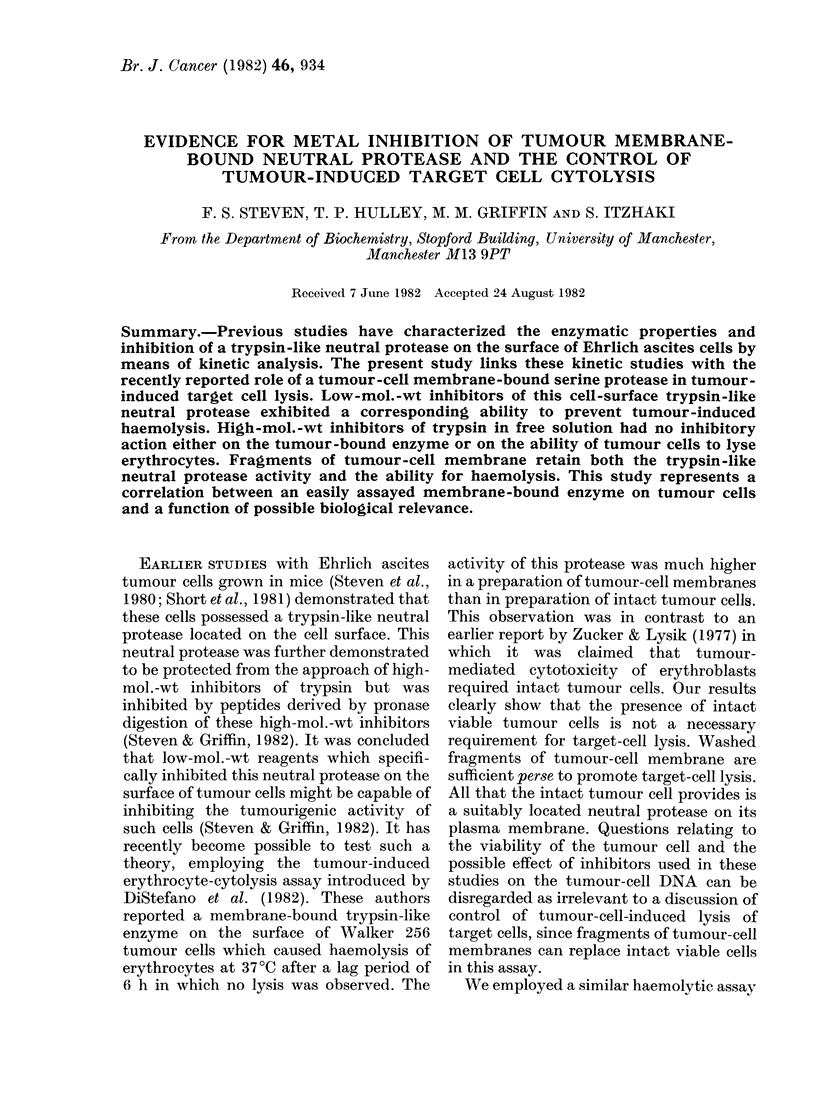

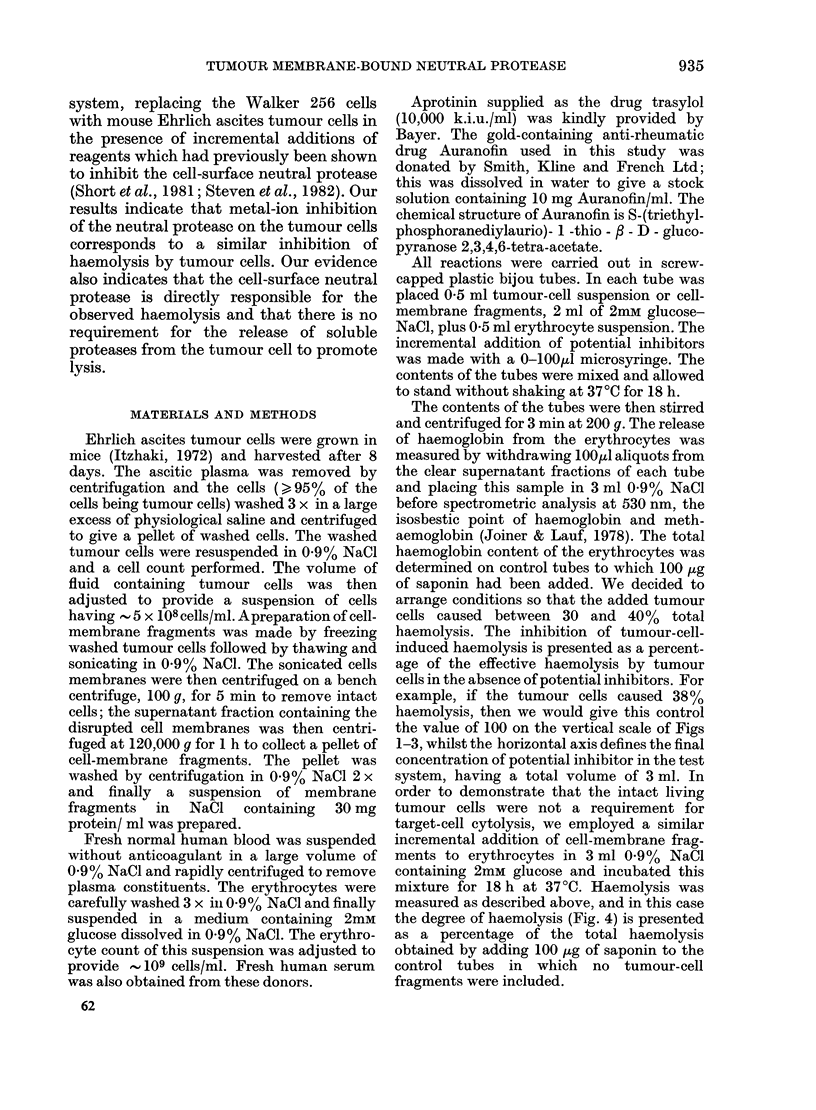

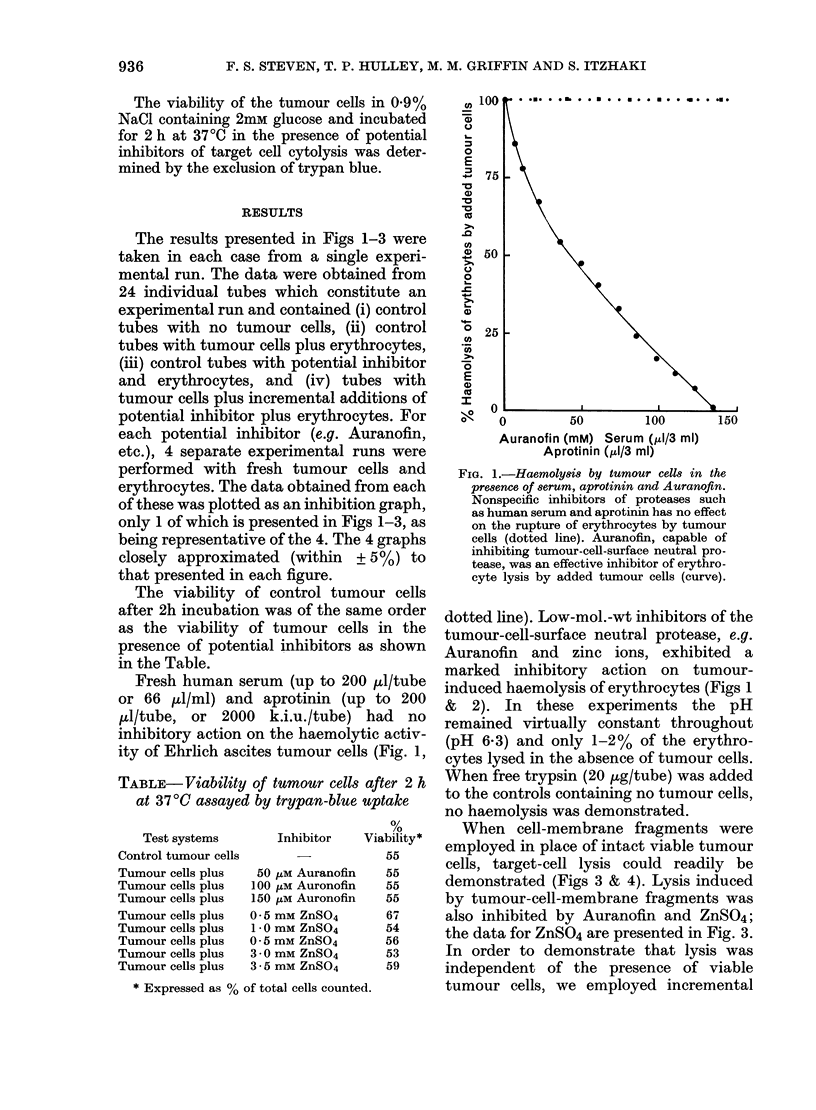

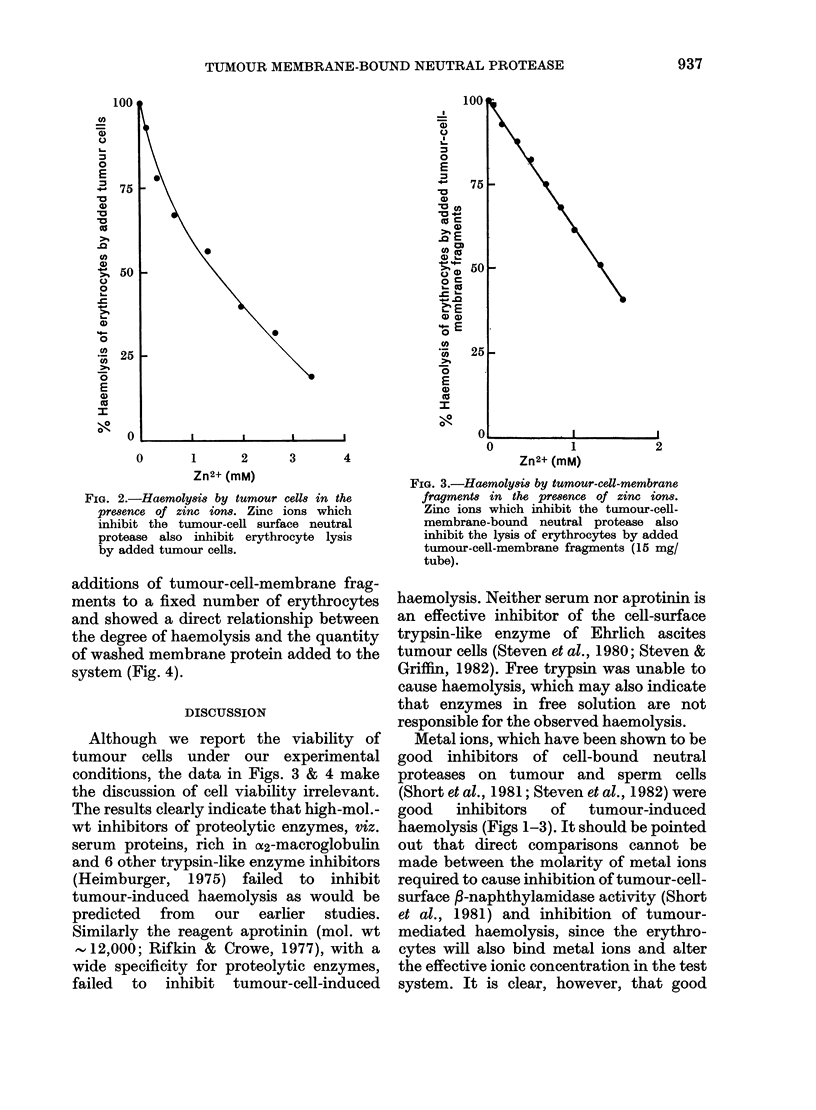

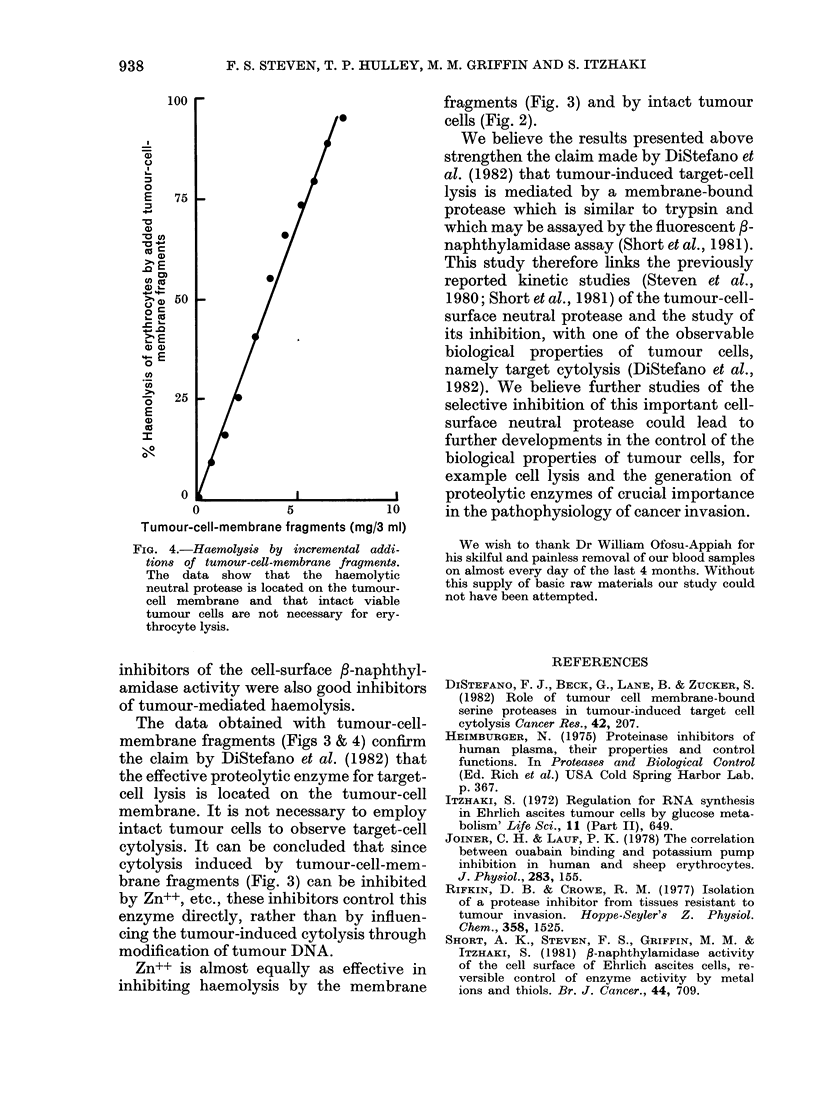

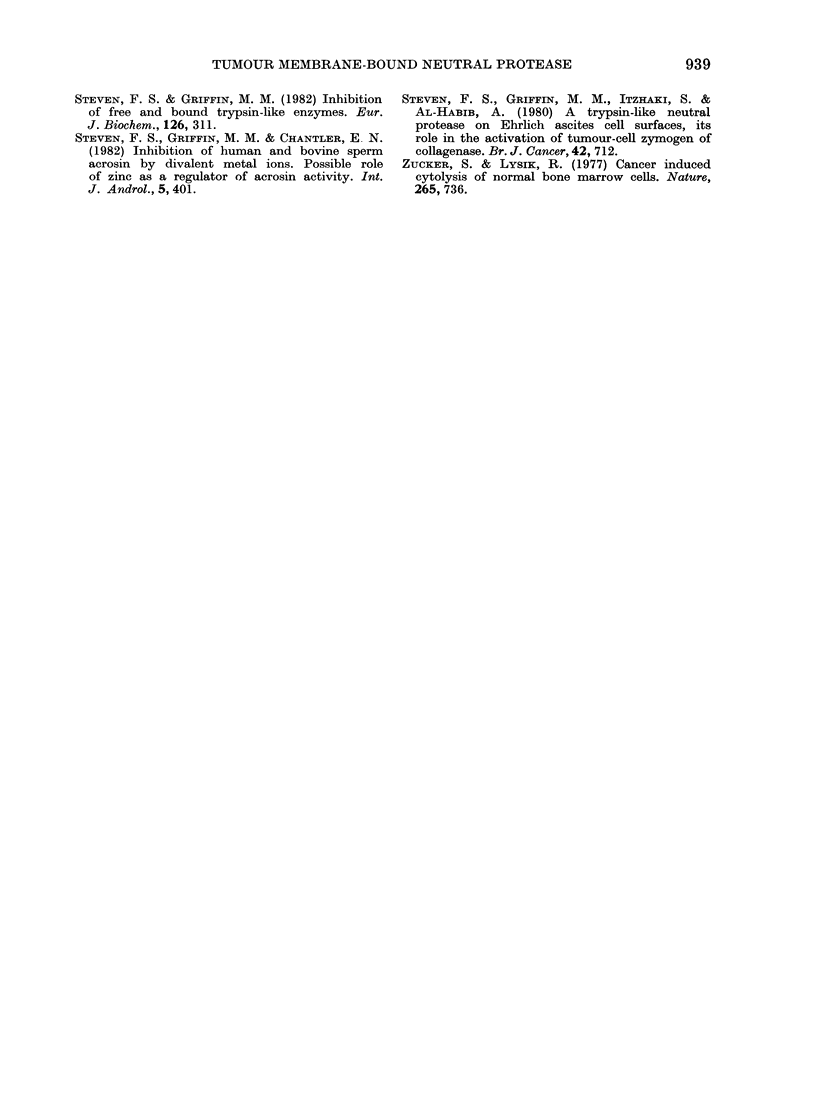


## References

[OCR_00579] DiStefano J. F., Beck G., Lane B., Zucker S. (1982). Role of tumor cell membrane-bound serine proteases in tumor-induced target cytolysis.. Cancer Res.

[OCR_00590] Itzhaki S. (1972). Regulation of uracil utilization for RNA synthesis in Ehrlich ascites tumour cells by glucose metabolism.. Life Sci II.

[OCR_00595] Joiner C. H., Lauf P. K. (1978). The correlation between ouabain binding and potassium pump inhibition in human and sheep erythrocytes.. J Physiol.

[OCR_00601] Rifkin D. B., Crowe R. M. (1977). Isolation of a protease inhibitor from tissues resistant to tumor invasion.. Hoppe Seylers Z Physiol Chem.

[OCR_00607] Short A. K., Steven F. S., Griffin M. M., Itzhaki S. (1981). beta-Naphthylamidase activity of the cell surface of Ehrlich ascites cells. Reversible control of enzyme activity by metal ions and thiols.. Br J Cancer.

[OCR_00621] Steven F. S., Griffin M. M., Chantler E. N. (1982). Inhibition of human and bovine sperm acrosin by divalent metal ions. Possible role of zinc as a regulator of acrosin activity.. Int J Androl.

[OCR_00628] Steven F. S., Griffin M. M., Itzhaki S., Al-Habib A. (1980). A trypsin-like neutral protease on Ehrlich ascites cell surfaces: its role in the activation of tumour-cell zymogen of collagenase.. Br J Cancer.

[OCR_00616] Steven F. S., Griffin M. M., Itzhaki S. (1982). Inhibition of free and bound trypsin-like enzymes.. Eur J Biochem.

[OCR_00635] Zucker S., Lysik R. (1977). Cancer-induced cytolysis of normal bone marrow cells.. Nature.

